# Delirium in Critically Ill Patients with and without COVID-19—A Retrospective Analysis

**DOI:** 10.3390/jcm10194412

**Published:** 2021-09-26

**Authors:** Markus Jäckel, Nico Aicher, Paul Marc Biever, Laura Heine, Xavier Bemtgen, Jonathan Rilinger, Viviane Zotzmann, Alexander Supady, Peter Stachon, Tobias Wengenmayer, Christoph Bode, Dawid Leander Staudacher

**Affiliations:** 1 Department of Cardiology and Angiology I, Heart Center Freiburg University, Faculty of Medicine, University of Freiburg, 79106 Freiburg, Germany; nico.aicher@uniklinik-freiburg.de (N.A.); paul.biever@uniklinik-freiburg.de (P.M.B.); laura.heine@uniklinik-freiburg.de (L.H.); xavier.bemtgen@uniklinik-freiburg.de (X.B.); jonathan.rilinger@uniklinik-freiburg.de (J.R.); viviane.zotzmann@uniklinik-freiburg.de (V.Z.); alexander.supady@uniklinik-freiburg.de (A.S.); peter.stachon@uniklinik-freiburg.de (P.S.); tobias.wengenmayer@uniklinik-freiburg.de (T.W.); christoph.bode@uniklinik-freiburg.de (C.B.); dawid.staudacher@uniklinik-freiburg.de (D.L.S.); 2 Department of Medicine III (Interdisciplinary Medical Intensive Care), Medical Center, Faculty of Medicine, University of Freiburg, 79106 Freiburg, Germany

**Keywords:** delirium, COVID-19, SARS-CoV-2, intensive care unit, Nudesc

## Abstract

Background: Delirium complicating the course of Intensive care unit (ICU) therapy is a known driver of morbidity and mortality. It has been speculated that infection with the neurotrophic SARS-CoV-2 might promote delirium. Methods: Retrospective registry analysis including all patients treated at least 48 h on a medical intensive care unit. The primary endpoint was development of delirium as diagnosed by Nursing Delirium screening scale ≥2. Results were confirmed by propensity score matching. Results: 542 patients were included. The primary endpoint was reached in 352/542 (64.9%) patients, without significant differences between COVID-19 patients and non-COVID-19 patients (51.4% and 65.9%, respectively, *p* = 0.07) and correlated with prolonged ICU stay in both groups. In a subgroup of patients with ICU stay >10 days delirium was significantly lower in COVID-19 patients (*p* ≤ 0.01). After adjustment for confounders, COVID-19 correlated independently with less ICU delirium (*p* ≤ 0.01). In the propensity score matched cohort, patients with COVID-19 had significantly lower delirium incidence compared to the matched control patients (*p* ≤ 0.01). Conclusion: Delirium is frequent in critically ill patients with and without COVID-19 treated at an intensive care unit. Data suggests that COVID-19 itself is not a driver of delirium per se.

## 1. Introduction

In 2019 the novel severe acute respiratory syndrome coronavirus-2 (SARS-CoV-2) emerged and evolved to a pandemic that challenges health care professionals worldwide. The clinical symptoms of coronavirus disease 2019 (COVID-19) vary from asymptomatic patients via fever and cough through to severe pneumonia and life-threatening acute respiratory distress syndrome (ARDS) [1,2,3]. Up to one-fourth of all hospitalized patients suffering from COVID-19 can be classified as critically ill, requiring intensive care treatment including invasive mechanical ventilation or vasopressor-therapy [4,5].

Despite the respiratory pathologies, SARS-CoV-2 has been shown to be associated with neurological symptoms and delirium [6]. Several pathophysiological pathways including direct neuro-invasiveness, vascular alterations causing local hypoxic damage, and systemic inflammation processes resulting in cytokine induced damage to brain cells are discussed [7,8]. Even though the pathophysiology of delirium is still poorly understood, neuroinflammation caused by inflammatory mediators and neurotransmitter disturbances induced by hypoxia are potential causes for neurocognitive impairment such as delirium [9,10]. These parallels in pathophysiologic hypotheses trigger new research interests in SARS-CoV-2 and causes for delirium.

It is still debatable if SARS-CoV-2 is specifically associated with delirium. We have recently shown that there is no significant difference regarding delirium characteristics in patients with SARS-CoV-2 or influenza [11]. Based on this, we compared COVID-19 patients and non-COVID-19 patients on our medical intensive care unit (ICU) to identify, whether COVID-19 is a specific risk factor for development of delirium. As secondary endpoints, known predictors and outcomes of delirium were investigated in this dataset in order to verify the validity of the delirium diagnosis used in this research.

## 2. Materials and Methods

We conducted an investigator-initiated single-center retrospective cohort study analyzing patients from the Freiburg COVID-19 registry treated from March 2020 until May 2021. This registry included all patients with reverse transcriptase polymerase chain reaction (rtPCR)-confirmed SARS-CoV-2 treated on our ICU. Patients from the Freiburg delirium registry treated in 2019 were evaluated as a control group.

Analysis was blinded to patient identity and was covered by an ethics approval (Ethics Committee of Albert-Ludwigs-University of Freiburg, file number 387/19). All methods were performed in accordance with the relevant guidelines and regulations. Since only retrospective data of an already performed intervention was collected, informed consent was waived by the approval of the relevant ethic committee.

### 2.1. Patient Selection and Data Collection

All patients treated at the Interdisciplinary Medical Intensive Care Unit (MIT) at the Medical Center, University of Freiburg, Germany for at least 48 h were included in the present analysis. In cases of readmission, only the index ICU stay was considered. Exclusion criteria included all conditions that made a delirium evaluation impossible. Practically, patients that were intubated during the whole clinical course as well as patients with severe neurologic comorbidities or severe hypoxic brain dysfunction and who therefore could not be evaluated for delirium even once were excluded.

All outcome variables were evaluated by manual case-by-case review of medical and patient records. Only the ICU stay was analyzed. Since only data from the index hospital stay was evaluated, no patients were lost to a follow up. The registry was checked for data integrity and plausibility according to the RECORD recommendations for data clearing [12]. Research is presented according to the STROBE guidelines for reporting observational studies [13].

### 2.2. Definition of Delirium

Delirium is a common complication in daily practice on our ICU. According to local standard operating procedures, efforts are taken with respect to any patient in order to prevent and treat delirium with an interdisciplinary team approach including nurses, physiotherapists and physicians. Delirium was defined by a Nursing Delirium screening scale (Nudesc) ≥2 in at least one assessment according to Gaudreau et al. [14]. The Nudesc is routinely assessed by specially trained nurses for all patients on our ICU at least three times a day. The NuDesc is approved, easy to use and has a reported sensitivity (93–98%) and specificity (81–87%) for diagnosis [14,15,16]. Delirium positive days were defined as the ratio of days with a positive delirium assessment to all days on which an assessment was possible.

The motoric subtype of delirium was defined using the Richmond agitation and sedation scale (RASS), which is assessed at least three times daily as part of the daily routine on our ICU [17]. According to the literature, hyperactive delirium was presumed when diagnosed in conjunction with RASS ≥1 and no RASS <0 in follow-up scores during delirium [18]. RASS scores <0 after necessary sedation due to agitation were excluded. Hypoactive delirium was presumed diagnosed in the context of an RASS ≤0, whereas mixed delirium was defined as variable positive and negative RASS.

### 2.3. Bias

Bias was reduced by predefining the primary endpoint “delirium” using a well-established score. Interpretation of variables was minimized and clear cutoff values were predefined. An adjustment for confounders was done by multivariate logistic regression analysis and propensity score matching.

### 2.4. Statistical Methods

All relevant data is given in standardized tables. For data analysis, SPSS (version 26, IBM Statistics, Armonk, NY, USA) and Prism (version 8, GraphPad, San Diego, CA, USA) were employed. For statistical analysis the Mann–Whitney U-test was used for analysis of continuous variables, including length of ICU stay. For categorical variables, Fisher’s exact test was used when the number of expected values was smaller than five; otherwise, Pearson’s Chi-square test was performed. A *p*-value of <0.05 was considered statistically significant.

In order to identify the impact of different variables, subgroup analyses were performed. In order to estimate the impact of COVID-19, a binary multivariable regression analysis was performed. We incorporated only predictors where COVID-19 and non-COVID-19 patients differed significantly and predictors of delirium which are known to significantly differentiate between patients with and without delirium (forward selection process with a *p*-value threshold of 0.01). Again, a *p*-value of <0.05 was considered statistically significant. Propensity score matching was performed using SPSS with a nearest neighbor matching algorithm using a caliper of 0.2. Matching was performed for age, duration of ICU stay, necessity of non-invasive or invasive ventilation, dementia, and alcohol abuse. Data are given as *n* (%), median and interquartile range (25th–75th) or odds ratio (OR) with a 95% confidence interval (CI) if not stated otherwise.

## 3. Results

### 3.1. Study Population

**Figure 1:**
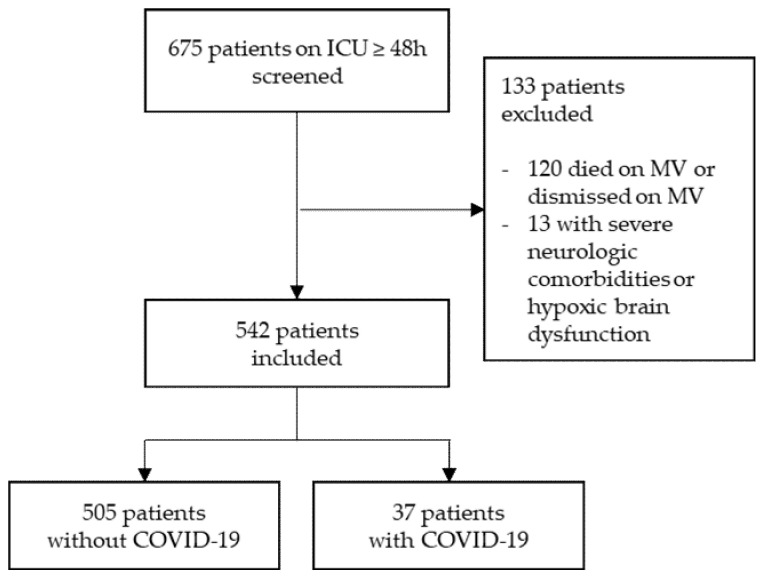
Study population. Flow chart showing patients treated on a medical ICU for at least 48 h that were screened for inclusion. Patients dismissed on invasive mechanical ventilation (MV) without tracheostomy and who therefore could not be evaluated precisely for delirium and patients with severe neurological diseases had to be excluded.

Of the screened 675 patients, 584 were severe acute respiratory syndrome coronavirus 2 (SARS-CoV-2) negative (“Non-COVID-19”) and 91 were positive (“COVID-19”). Of these, 133 (79/54) patients were excluded. Specifically, 120 (66/54) patients died on invasive mechanical ventilation or were transferred to other hospitals before extubation, and 13 (13/0) had severe neurological comorbidities or hypoxic brain dysfunction (Figure 1).

A total of 542 patients were included. The mean age was 69.3 (58.1–79.0) years and 197/542 (36.3%) were female. Of all included patients, 505/542 were COVID-19 SARS-CoV-2 negative and 37/542 were positive. COVID-19 patients were younger: 61.0 (47.5–72.0) versus 69.5 (58.5–79.0) years; *p* = 0.002. No significant differences were identified concerning comorbidities (Table 1).

The initial cause for ICU treatment was more often for respiratory reasons in COVID-19 patients than in non-COVID-19 patients (29 (78.4%) versus 122 (24.2%); *p* < 0.001). COVID-19 patients more often had non-invasive and invasive ventilation and venovenous extracorporeal membrane oxygenation (V-V ECMO) organ support. ICU stays were significantly longer in COVID-19 patients (10.6 (4.9–18.7) versus 4.7 (2.9–8.1) days; *p* < 0.001) (Table 2).

### 3.2. Delirium

Delirium was detected in 352/542 (64.9%) patients. No significant differences were seen concerning delirium incidence in COVID-19 patients (19/37 (51.4%)) and non-COVID-19 patients (333/505 (65.9%); *p* = 0.073). No significant differences were seen concerning delirium positive days (Table 2). Delirium onset, highest Nudesc, duration of delirium as well as delirium presentation did not differ significantly (Table 3).

**Figure 2:**
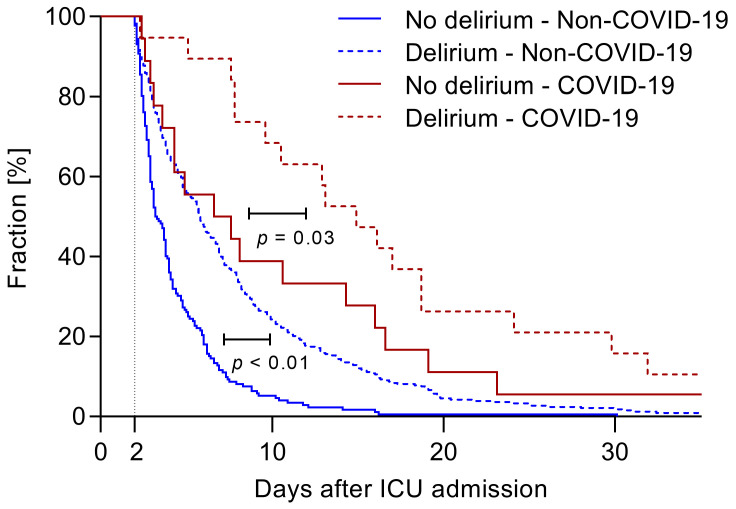
Duration of stay on the ICU. Graph showing ICU stay of all patients included, separated by the presence of COVID-19 and incidence of delirium.

The length of ICU stay was significantly longer in COVID-19 (delirium: 14.9 (7.8–24.11) days; no delirium 7.1 (3.5–16.2) days; *p* = 0.031) and non-COVID-19 (delirium: 5.8 (3.3–9.8) days; no delirium: 3.3 (2.6–5.2) days; *p* < 0.001) patients (Figure 2).

### 3.3. Subgroup Analysis

**Figure 3:**
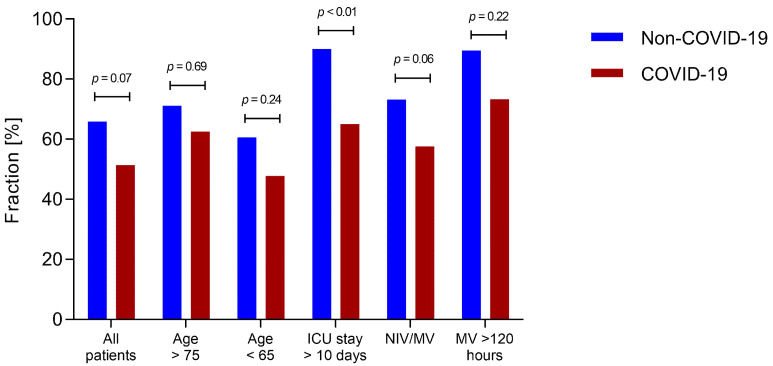
Fraction of delirium positive patients in different subgroups. Graph shows the fraction of patients with delirium as diagnosed by Nudesc in COVID-19 and non-COVID-19 patients in different subgroups. NIV: non-invasive ventilation; IV: Invasive ventilation.

Considering the significant differences in age, duration of ICU stay, and non-invasive and invasive ventilation seen between the COVID-19 and non-COVID-19 patients, subgroup analyses were performed. When analyzing only patients with an ICU stay of more than 10 days (COVID-19: *n* = 20/non-COVID-19: *n* = 90) delirium occurred significantly more often in the non-COVID-19 group. In patients with non-invasive or invasive ventilation delirium incidence did not differ significantly, although there was a tendency to increased delirium incidence in non-COVID-19 patients (*n* = 33/298; *p* = 0.06). No significant differences were seen in subgroups of patients older than 75, patients younger than 65 and patients with mechanical ventilation for more than 120 h. (*n* = 8/187: 23/193; 18/61) (Figure 3).

### 3.4. Association of COVID-19 and Delirium

**Figure 4:**
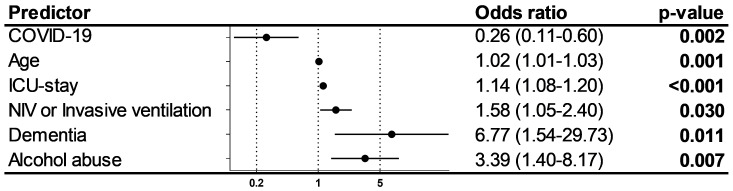
Predictors of delirium. Figure shows multivariable logistic regression analysis with odds ratio (95% confidence interval) of different predictors for delirium diagnosed by Nudesc. Odds ratios >1 mark positive predictors, odds ratio <1 negative predictors.

As the two groups were heterogenous, a multivariable binary logistic regression analysis was performed to clarify the association of COVID-19 and delirium. When analyzed with age, duration of ICU stay, necessity of non-invasive or invasive ventilation, dementia, and alcohol abuse, COVID-19 showed an independent protective effect concerning the appearance of delirium (OR 0.26 (0.11–0.60); *p* = 0.002). Age, duration of ICU stay, necessity of non-invasive or invasive ventilation, dementia, and alcohol abuse were positively associated with delirium (Figure 4).

**Figure 5:**
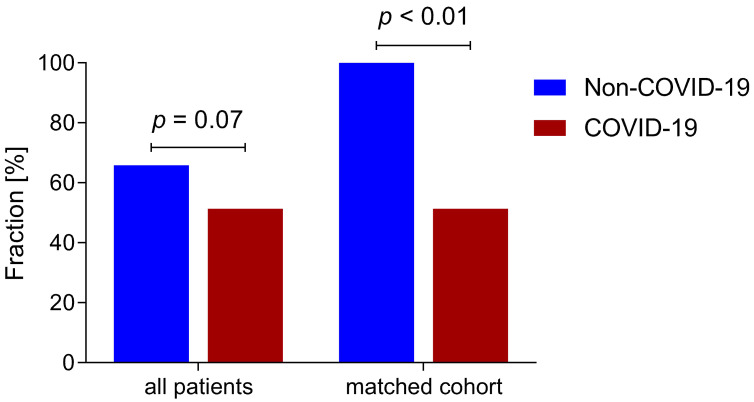
Delirium incidence. Graph shows delirium incidence in all patients and a propensity score matched cohort of COVID-19 and non-COVID-19 patients.

Propensity score matching was performed. Seventy-three propensity score matched patients (37 COVID-19 patients, 36 non-COVID-19 patients) with similar baseline characteristics were analyzed. Delirium incidence was significantly lower in the COVID-19 group compared to the non-COVID-19 group (51.4% versus 100%; *p* < 0.001) (Figure 5).

## 4. Discussion

The primary endpoint of this study (delirium on a medical ICU) in the whole cohort was detected in ~65%.

This rate is well in line with literature reporting incidence of delirium in medical ICU patients in 19–82%, depending on setting and mode of detection [19,20,21,22,23,24,25,26,27]. A multicenter cohort study showed a delirium rate of ~55% in COVID-10 patients with ARDS, which is close to the rate we showed for COVID-19 patients (51%) [28]. Other relevant characteristics such as age and SAPS2 were also similar to those in the referred study, showing the representativity of our results. The Nudesc score, used to diagnose delirium in our study, is a validated tool with high specificity for delirium [14,15,16]. Known predictors of delirium (age, dementia, alcohol abuse) correlated nicely with delirium detected in this registry and patients with delirium had longer ICU stays. These facts suggest the validity of the endpoint used.

Comparing the incidence of delirium in patients with and without COVID-19 in patients treated at a medical ICU, we found similar rates of delirium in both groups. After adjustment for confounders, a lower rate of delirium in COVID-19 was detected compared to the whole cohort. This finding, confirmed by propensity score matching, suggests that delirium was less frequent in patients with COVID-19 compared to those without.

At first glance, these findings might seem counterintuitive since SARS-CoV-2 has neurotropic characteristics and might invade the central nervous system via the angiotensin converting enzyme 2 receptors expressed in the olfactory bulb, thereby causing neuroinflammation and ultimately delirium [29]. It is important, however, to consider that several other medical conditions like acute respiratory distress syndrome [27], cardiac arrest [30], and acute kidney injury [31] might also complicate the ICU course of COVID-19 patients. Our data suggest that known promoters of delirium including age, duration of ICU stay, dementia, alcohol abuse, and disease severity trigger delirium rather that SARS-CoV-2 infection. These findings, however, should not obscure the fact that delirium incidence was still more than 50% in COVID-19 patients in our sample, and was reported to be up to 84% in the literature [6]. All available measures should be undertaken in order to limit the burden of delirium in these vulnerable patient groups [32].

### Limitations

When discussing results, some limitations have to be considered. First of all, delirium was assessable in only a small sample size of COVID-19 patients compared to non-COVID-19 patients. Nevertheless, results showed clearly significant differences concerning delirium incidence in a propensity score matched cohort. Since one positive delirium assessment was considered as “delirium positive” we cannot exclude false positive assessments in patients awakening from sedation. As clinical data was based on medical reports, some variables may be underreported and some confounders may not have been measured. Finally, we present single-center retrospective data and results have to be considered hypotheses-generating.

## 5. Conclusions

Delirium is frequent in critically ill patients treated at an intensive care unit with and without COVID-19. Known risk factors promote the development of delirium including age, duration of ICU stay, dementia, alcohol abuse, and disease severity. Our data suggests that COVID-19 itself is not an independent driver of delirium per se.

## Figures and Tables

**Table 1:** Baseline Characteristics.

Baseline Characteristics	COVID-19 (*n* = 37)	Non-COVID-19 (*n* = 505)	*p*-Value
Age	61.0 (47.5–72.0)	69.5 (58.5–79.0)	0.002
Female	11 (29.7%)	186 (36.8%)	0.386
Comorbidities			
Heart rhythm disturbances	8 (21.6%)	141 (27.9%)	0.407
Coronary heart disease	9 (24.3%)	130 (25.7%)	0.849
Obesity	7 (18.9%)	60 (11.9%)	0.200
Pulmonary disease	6 (16.2%)	101 (20.0%)	0.577
Liver disease	1 (2.7%)	46 (9.1%)	0.237
Chronic kidney disease	7 (18.9%)	104 (20.6%)	0.807
Peripheral/cerebral arterial occlusive disease	2 (5.4%)	59 (11.7%)	0.415
Neurologic disease	4 (10.8%)	122 (24.2%)	0.064
Malignancy	5 (13.5%)	83 (16.4%)	0.642
Psychiatric disease	2 (5.4%)	56 (11.1%)	0.410
Dementia	0 (0.0%)	28 (5.5%)	0.246
Alcohol abuse	0 (0.0%)	45 (8.9%)	0.062
Drug abuse	0 (0.0%)	18 (3.6%)	0.626

Data given as absolute numbers *n* (% of all patients) or median and interquartile range (25th–75th). *p*-value reported in bold if difference is significant (*p* < 0.05).

**Table 2:** Clinical Characteristics.

Clinical Characteristics	COVID-19 (*n* = 37)	Non-COVID-19 (*n* = 505)	*p*
Delirium (NuDESC ≥ 2)	19 (51.4%)	333 (65.9%)	0.073
Delirium positive days (%)	4.4 (0–65.2)	39.1 (0–83.9)	0.068
ICU stay (days)	10.6 (4.9–18.7)	4.7 (2.9–8.1)	<0.001
Mortality	7 (18.9%)	70 (13.9%)	0.395
TISS 10 *	10 (10–15)	10 (5–15); *n* = 503	0.270
SAPS2 *	43 (30–47)	43 (34–52); *n* = 503	0.214
Non-invasive ventilation	28 (75.7%)	202 (40.0%)	<0.001
Invasive ventilation	18 (48.6%)	187 (37.0%)	0.159
Non-invasive or invasive ventilation	33 (89.2%)	298 (59.0%)	<0.001
Days on ventilation **	14.3 (6.0–17.6)	5.6 (2.3–9.3)	0.006
V-V ECMO	5 (13.5%)	10 (2.0%)	0.002
Catecholamine therapy	25 (67.6%)	294 (58.2%)	0.265
Norepinephrine	25 (67.6%)	277 (54.9%)	0.133
Dobutamine	3 (8.1%)	38 (7.5%)	0.753
Vasopressin	2 (5.4%)	22 (4.4%)	0.675
Renal replacement therapy	5 (13.5%)	63 (12.5%)	0.798
Necessity of blood transfusions	16 (43.2%)	177 (35.0%)	0.315
Cause of illness			
Respiratory	29 (78.4%)	122 (24.2%)	<0.001
Cardiac	7 (18.9%)	254 (50.3%)	<0.001
Septic	1 (2.7%)	96 (19.0%)	0.012
Other	0 (0.0%)	79 (15.6%)	0.009

Data given as absolute numbers *n* (% of all patients) or median and interquartile range (25th–75th). *p*-value reported in bold if difference is significant (*p* < 0.05). * at admission; ** only patients with invasive ventilation included. TISS 10: Therapeutic Intervention Scoring System 10; SAPS2: Simplified Acute Physiology Score 2; V-V ECMO: venovenous extracorporeal membrane oxygenation.

**Table 3:** Delirium and Outcomes.

	COVID-19 (*n* = 19)	Non-COVID-19 (*n* = 333)	*p*
Delirium onset (days) *	1 (0–2)	0 (0–1)	0.260
Highest NuDESC	4 (3–5)	4 (3–6)	0.726
Duration of delirium (days)	3 (1–7)	3 (1–5)	0.643
Hypoactive delirium	6 (31.6% **)	104 (31.2% **)	0.975
Mixed delirium	8 (42.1% **)	169 (50.8% **)	0.464
Hyperactive delirium	5 (26.3% **)	60(18.0% **)	0.365

Data given as absolute numbers *n* (% of all patients) or median and interquartile range (25th–75th). *p*-value reported in bold if difference is significant (*p* < 0.05). * after first possible delirium assessment; ** percentage of delirious patients in each subgroup.

## Data Availability

The datasets used and analyzed during the current study are available from the corresponding author on reasonable request.
